# Refractory ineffective triggering during pressure support ventilation: effect of proportional assist ventilation with load-adjustable gain factors

**DOI:** 10.1186/s13613-021-00935-0

**Published:** 2021-10-20

**Authors:** Anne-Fleur Haudebourg, Tommaso Maraffi, Samuel Tuffet, François Perier, Nicolas de Prost, Keyvan Razazi, Armand Mekontso Dessap, Guillaume Carteaux

**Affiliations:** 1 Service de Médecine Intensive Réanimation, DHU A-TVB, Hôpitaux Universitaires Henri Mondor – Albert Chenevier, Assistance Publique – Hôpitaux de Paris (AP-HP), Créteil, France; 2 Groupe de Recherche Clinique CARMAS, IMRB, Faculté de Médecine de Créteil, Université Paris Est-Créteil, Créteil, France; 3 Institut Mondor de Recherche Biomédicale INSERM 955, Créteil, France; 4 Service de Réanimation et Surveillance Continue Adulte, Centre hospitalier intercommunal de Créteil, 94000 Créteil, France

**Keywords:** Mechanical ventilation, Patient, Ventilator asynchrony, Ineffective triggering, Proportional assist ventilation

## Abstract

**Background:**

Ineffective triggering is frequent during pressure support ventilation (PSV) and may persist despite ventilator adjustment, leading to refractory asynchrony. We aimed to assess the effect of proportional assist ventilation with load-adjustable gain factors (PAV+) on the occurrence of refractory ineffective triggering.

**Design:**

Observational assessment followed by prospective cross-over physiological study.

**Setting:**

Academic medical ICU.

**Patients:**

Ineffective triggering was detected during PSV by a twice-daily inspection of the ventilator’s screen. The impact of pressure support level (PSL) adjustments on the occurrence of asynchrony was recorded. Patients experiencing refractory ineffective triggering, defined as persisting asynchrony at the lowest tolerated PSL, were included in the physiological study.

**Interventions:**

Physiological study: Flow, airway, and esophageal pressures were continuously recorded during 10 min under PSV with the lowest tolerated PSL, and then under PAV+ with the gain adjusted to target a muscle pressure between 5 and 10 cmH_2_O.

**Measurements:**

Primary endpoint was the comparison of asynchrony index between PSV and PAV+ after PSL and gain adjustments.

**Results:**

Among 36 patients identified having ineffective triggering under PSV, 21 (58%) exhibited refractory ineffective triggering. The lowest tolerated PSL was higher in patients with refractory asynchrony as compared to patients with non-refractory ineffective triggering. Twelve out of the 21 patients with refractory ineffective triggering were included in the physiological study. The median lowest tolerated PSL was 17 cmH_2_O [12–18] with a PEEP of 7 cmH_2_O [5–8] and FiO_2_ of 40% [39–42]. The median gain during PAV+ was 73% [65–80]. The asynchrony index was significantly lower during PAV+ than PSV (2.7% [1.0–5.4] vs*.* 22.7% [10.3–40.1], *p* < 0.001) and consistently decreased in every patient with PAV+. Esophageal pressure–time product (PTPes) did not significantly differ between the two modes (107 cmH_2_O/s/min [79–131] under PSV vs*.* 149 cmH_2_O/s/min [129–170] under PAV+, *p* = 0.092), but the proportion of PTPes lost in ineffective triggering was significantly lower with PAV+ (2 cmH_2_O/s/min [1–6] vs*.* 8 cmH_2_O/s/min [3–30], *p* = 0.012).

**Conclusions:**

Among patients with ineffective triggering under PSV, PSL adjustment failed to eliminate asynchrony in 58% of them (21 of 36 patients). In these patients with refractory ineffective triggering, switching from PSV to PAV+ significantly reduced or even suppressed the incidence of asynchrony.

**Supplementary Information:**

The online version contains supplementary material available at 10.1186/s13613-021-00935-0.

## Background

Ineffective triggering is the most frequent asynchrony during pressure support ventilation (PSV) [1–3] and is associated with poor outcome [1, 2, 4]. Dynamic hyperinflation is the main pathophysiological mechanism underlying its occurrence [1, 5]. Such dynamic hyperinflation may arise when increasing the pressure support level (PSL) and ineffective effort is therefore usually considered as a sign of over-assistance [6–10]. Indeed, the most efficient ventilator’s setting adjustment to reduce the incidence of ineffective triggering is to decrease the PSL [11]. In some patients exhibiting a high incidence of ineffective triggering, however, decreasing the PSL leads to the appearance of signs of poor tolerance, as respiratory distress or dyspnea, without suppressing asynchrony [11]. These patients can be considered as experiencing refractory asynchrony under PSV. The incidence of refractory ineffective triggering is currently unknown.

Proportional assist ventilation with load-adjustable gain factors (PAV+) is a ventilatory mode that delivers assistance in proportion to the instantaneous flow and volume, calculating the instantaneous pressure needed to overcome the elastic and resistive pressures [12–14]. Assistance, called the gain, is expressed as a percentage of the total pressure needed to inflate the respiratory system and is adjusted by the clinician. Thus, during PAV+, assistance is directly in proportion to the patient’s inspiratory effort [12]. It is therefore feasible to adjust the gain in order to maintain the patient within a desirable range of inspiratory effort [15], which should theoretically avoid over- or under-assistance. Furthermore, gain adjustments have little influence on tidal volume [10] and ventilator’s insufflation time [10, 16], which strongly limits the occurrence of dynamic hyperinflation and therefore of ineffective triggering [10, 16–19].

Two physiological studies comparing PAV+ to PSV without specific PSL optimization reported a lower incidence of patient–ventilator asynchronies with PAV+ [16, 18]. The PSL, however, was much higher (up to 29 cmH_2_O (18)) than what has been reported as the optimal level of assistance in patients exhibiting a high incidence of ineffective efforts (13 cmH_2_O as a median [11]. Thus, the benefit of using PAV+ in patients experiencing refractory ineffective triggering during PSV is unknown. We hypothesized that PAV+ may reduce the incidence of such refractory asynchronies.

The main aim of our study was therefore to assess the effect of PAV+ on the incidence of ineffective triggering in patients exhibiting refractory ineffective efforts during PSV.

## Materials and methods

This was a prospective study, conducted over a 16-month period in the Henri Mondor University Hospital Medical ICU, Créteil, France. The observational part of this study was approved by the ethics committee of the Société de Réanimation de Langue Française (French Intensive Care Society) and the physiological part by the ethics committee “CPP Région Centre—Ouest 1.” Written and oral information about this study was given to patients or families. Written consent was waived due to the observational nature of this study.

### Patients

In our unit, switching from assist control ventilation to PSV is attempted by the attending physician as soon as the patient meets the following criteria: ability to trigger each cycle of the ventilator, pulse oximetry greater than 90% with a FiO_2_ lower than 60%, no need for epinephrine or norepinephrine at a rate greater than 1 mg/h, and stable neurologic status with decreasing or no sedation [20, 21]. PSV is then continued in the absence of respiratory, hemodynamic, or neurologic deterioration. Patients under PSV were screened twice daily during 15 min every working day. Ineffective triggering was detected by visual inspection of the ventilator’s screen [11, 22]. In patients with a high incidence of ineffective triggering (more than 10% of the respiratory efforts [1, 3, 11]), the PSL was gradually decreased according to our usual practice in steps of 2 cmH_2_O until either ineffective triggering was eliminated or any predefined sign of poor respiratory tolerance occurred. Predefined signs of poor respiratory tolerance in our usual care procedure were the following: respiratory rate greater than 35/min, a drop in SpO_2_ below 90%, and sternocleidomastoid muscle activation [11]. If the decrease in PSL was well tolerated and led to the suppression of ineffective triggering, the patient was not eligible for the physiological study. If any of the predefined signs of poor tolerance arose during the stepwise decrease of the PSL before the suppression of ineffective triggering, the patient was eligible for this study. In that case, the PSL was immediately re-increased at its previous value, which corresponded to the minimal well-tolerated value and was considered as the lowest tolerated PSL. Non-inclusion criteria were age younger than 18 years, pregnancy, contraindication to esophageal catheter insertion, a need for FiO_2_ of at least 0.60 to maintain the SpO_2_ above 90%, hemodynamic instability requiring more than 1 mg/h of epinephrine or norepinephrine, severe central neurological disorders, agitation defined as a RASS score ≥ 2 [23], diaphragmatic paralysis, and chest tube with bronchopleural fistulae.

### Physiological study

#### Ventilator settings

Ventilators having PAV+ mode available were used (Puritan Bennett 980 or Puritan Bennett 840, Medtronic®). External positive end-expiratory pressure (PEEP) was maintained to the level previously set by the clinician. After inclusion, the patients were ventilated 20 min in PSV, then 20 min in PAV+. As these two modes operate in very different ways, we aimed to use methods for adjusting the level of assistance: (1) that optimize patient–ventilator interactions by taking into account the functioning of each mode; (2) while being simple, easy to implement and not requiring the use of advanced physiological tools (Pes), so that the results of the research can be more easily transferred into clinical routine.

During PSV, the PSL was maintained at its lowest tolerated value, as previously defined. The inspiratory trigger was set at 3 L/min and the cycling-off criterion at 25% of the peak inspiratory flow.

During PAV+, the gain (percentage of assistance) was initially set at 50% and then adjusted in steps of 5% to target a peak muscle pressure between 5 to 10 cmH_2_0 using the following equation [15] [Pmus_peak_ = Paw_peak_* − PEEP × (100 − Gain)/Gain*], where Pmus_peak_ is the peak muscle pressure of the respiratory muscles and Paw_peak_ the peak airway pressure. The first gain allowing reaching the peak muscle pressure target range was maintained during the 20-min period of PAV+ assessment. At the end of this study, the ventilation was resumed in PSV.

#### Measurements

The flow, airway, and esophageal pressure signals were recorded during the last 10 min of each 20-min period (see Additional file 1 for more details).

Ineffective efforts were identified from the combined analysis of the flow, airway, and esophageal pressures signals as previously described [1]. Their frequency was expressed as the asynchrony index [1]. The following measurements were averaged on fifteen cycles (Additional file 1: Figure S1): inspiratory delay was defined as the time between the onset of the decrease in esophageal pressure and the beginning of the ventilator’s insufflation; intrinsic PEEP was defined as the esophageal pressure drop during the inspiratory delay and could also capture expiratory muscles relaxation; insufflation time was defined as the time from the onset to the end of positive flow; tidal volume was obtained by integrating the flow signal during insufflation; esophageal pressure time product (PTPes) was computed as previously described [24].

### Endpoints

Primary endpoint was the comparison of asynchrony index between PSV set at the lowest tolerated PSL and PAV+ with the gain adjusted according to the Pmus_peak_. Secondary endpoints were as follows: (i) the clinical and physiological description of patients with refractory asynchronies; (ii) the effect of PAV + on tidal volume, ventilator’s respiratory rate, patient’s respiratory rate, triggering delay, auto-PEEP, insufflation time, Pmus_peak_, PTPes, PaO_2_/FiO_2,_ and PaCO_2_.

### Statistical analysis

All statistical analysis was performed using SPSS Base 20.0 statistical software package (SPSS, Chicago, IL). From the study by Thille et al*.* [11], we hypothesized that patients with refractory asynchronies may have a mean asynchrony index of 20%. To be clinically relevant, PAV+ must reduce the asynchrony index in these patients to less than 10%, a threshold widely considered to define a high incidence of asynchrony [1, 3, 11, 25, 26]. The sample size required to demonstrate a reduction in the asynchrony index from 20 to 9% with a standard deviation up to half of means, a type I error of 0.05, and a statistical power of 0.9 is eight patients. Since our assumptions for calculating the sample size were based on very limited data, we decided to include at least 12 patients. Continuous variables are expressed as the median [25th–75th percentiles] and compared using a Wilcoxon paired test for related measures. Categorical data, expressed as percentages, were compared using a Mc Nemar test for pairwise comparisons. A *p* value < 0.05 was considered statistically significant.

## Results

### Patients

**Fig. 1:**
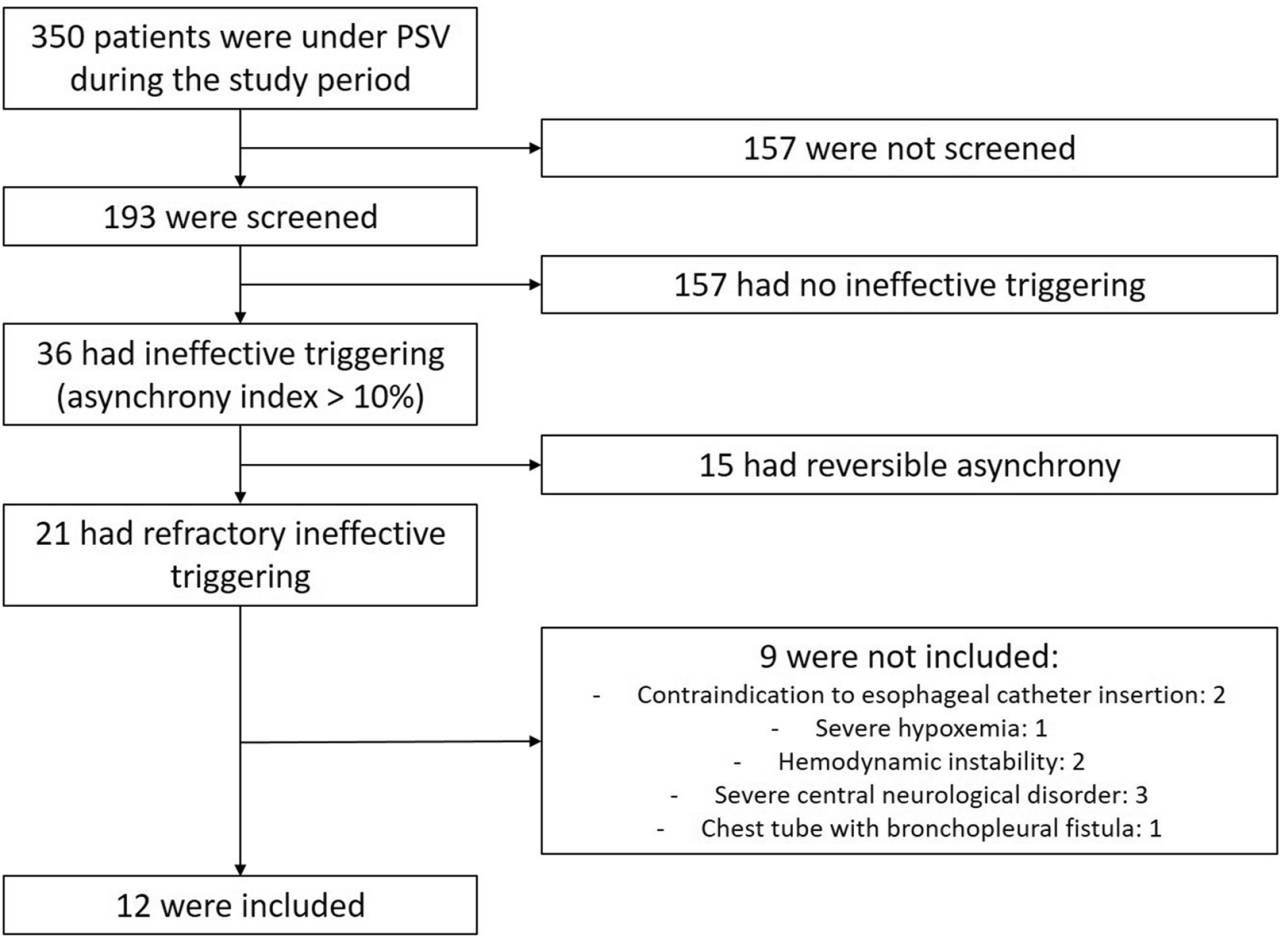
Flow chart. Flow chart of screening and inclusion. PSV denotes pressure support ventilation

Over a 16-month period, 415 screenings were performed in 193 out of the 350 patients ventilated in PSV (Fig. 1). Thirty-six patients (19%) exhibited ineffective triggering, among whom 21 (11%) experienced refractory ineffective triggering despite ventilator adjustment. Their demographic characteristics were not different from those of patients without refractory asynchrony (Table 1). However, their lowest tolerated PSL was significantly higher than that of patients with non-refractory ineffective triggering.

**Table 1:** Main characteristics of all screened patients

	All patients	Without ineffective triggering	With ineffective triggering			
			All	Non refractory	Refractory	
					All	Included
Number (% of all patients)	193 (100%)	157 (81%)	36 (19%)	15 (8%)	21 (11%)	12 (6%)
Age, years	64 [54–72]	65 [54–72]	64 [59–71]	65 [58–72]	64 [60–71]	63 [59–68]
Sex ratio, M/F	125/68	102/55	23/13	9/6	14/7	6/6
Weight, kg	75 [66–94]	75 [67–94]	78 [60–94]	79 [60–88]	77 [61–105]	83 [62–110]
Size, cm	170 [163–176]	170 [163–176]	171 [165–176]	169 [164–177]	172 [165–176]	171 [164–175]
BMI, kg/m^2^	26 [23–32]	26 [23–32]	25 [22–31]	25 [23–28]	25 [22–36]	31 [22–36]
COPD, n (%)	20 (10%)	14 (9%)	6 (17%)	2 (13%)	4 (19%)	3 (25%)
Initial PSL, cmH_2_O	10 [10–12]	10 [10–12]	14 [12–16]*	14 [11–14]	16 [13–18]	17 [14–18]
Optimized PSL, cmH_2_O	NA	NA	12 [10–15]	10 [8–13]	14 [12–18]**	17 [12–18]
PEEP, cmH_2_O	5 [5–7]	5 [5–7]	5 [5–8]	5 [5–6]	5 [5–8]	7 [5–8]
MV duration before screening, days	4 [2–7]	3 [2–7]	4 [3–12]	5 [2–12]	4 [3–9]	4 [3–7]

BMI: body mass index; COPD: chronic obstructive pulmonary disease; PSL: pressure support level; PEEP: positive end-expiratory pressure; MV: mechanical ventilation; NA: not applicable

*p < 0.05 vs*.* patients without ineffective triggering

**p < 0.05 vs*.* patients with non-refractory ineffective triggering

Nine out of the 21 patients with refractory ineffective triggering had non-inclusion criteria (contraindication to esophageal catheter, *n* = 2; need for FiO_2_ of at least 0.60 to maintain the SpO_2_ above 90%, *n* = 1; hemodynamic instability, *n* = 2; severe central neurological disorders, *n* = 3; and chest tube with bronchopleural fistulae, *n* = 1). Clinical and physiological characteristics of the 12 included patients are summarized in Tables 2 and 3, respectively. Only three of them had an underlying COPD, while most of them had a high respiratory rate (median of 41 [28–46] breaths/min) and needed a relatively high level of assistance (equal or above 16 cmH_2_O in 2/3 of them), with a respiratory effort within a normal range (median PTPes of 107 [79–131] cmH_2_O/s/min). FiO_2_ and PEEP were kept constant during this study. Under PAV+, the median gain was 73% [65–80].

**Table 2:** Main characteristics of the 12 included patients

Patient n°	Sex	Age (years)	BMI (kg/m^2^)	COPD	MV indication	SOFA at admission	MV duration before inclusion (days)	PSV duration before inclusion (days)	C_RS_ (mL/cmH_2_O)	R_RS_ (cmH_2_O/L/s)	SOFA at inclusion	PSL (cmH_2_O)	PEEP (cmH_2_O)	PEEPi (cmH_2_O)	MV duration (days)	Hospital survival
1	M	89	28	No	Coma	9	1	0	67	10	9	12	5	4.1	23	Yes
2	F	69	36	Yes	Sepsis	14	4	0	54	11	12	10	15	3.4	11	Yes
3	M	66	38	Yes	Cardiac arrest	10	3	0	24	17	8	18	8	8.5	10	Yes
4	M	50	41	No	AHF	9	8	2	23	11	6	16	6	4.2	9	Yes
5	F	60	34	No	Pneumonia	3	12	9	35	5	1	12	5	4.1	17	Yes
6	F	61	18	Yes	COPD exacerbation	10	4	1	81	13	6	18	8	13.7	12	Yes
7	F	63	38	No	Coma	6	28	27	29	3	5	18	8	2.9	165	No
8	F	55	22	No	Cardiac arrest	5	4	0	48	10	2	8	5	3.7	4	Ye
9	M	46	23	No	Other	8	2	0	22	6	6	18	5	1.9	5	Yes
10	F	70	23	No	Sepsis	12	7	4	22	16	7	18	8	0.2	10	Yes
11	M	63	36	No	Pneumonia	3	2	0	34	5	3	16	5	3.0	38	Yes
12	M	68	20	No	Pneumonia	12	4	0	68	5	10	18	8	4.1	7	No

BMI: body mass index; COPD: chronic obstructive pulmonary disease; MV: mechanical ventilation; SOFA: sepsis-related organ failure assessment; PSV: pressure support ventilation; C_RS_: respiratory system compliance; R_RS_: respiratory system resistance. CRS and RRS were recorded during PAV+. PSL: pressure support level; PEEP: positive end-expiratory pressure; PEEPi: intrinsic positive end-expiratory pressure; AHF: acute heart failure

**Table 3:** Physiological measurements under PSV and PAV+

	PSV	PAV+	Δ (PAV+ – PSV)	*p*
SpO_2_, %	96 [93–97]	96 [94–98]	− 1 [− 1–0]	0.629
PaO_2_/FiO_2_, mm Hg	228 [209–257]	213 [203–298]	− 14 [− 28–60]	0.820
PaCO_2_, mm Hg	36 [33–41]	45 [34–55]	8 [1–12]	0.032
Heart rate, /min	103 [89–109]	101 [96–108]	2 [− 1–8]	0.181
MAP, mm Hg	89 [79–94]	92 [88–98]	5 [0–12]	0.032
Lactate, mmol/L	1 [0.9–1.1]	0.9 [0.8–1]	− 0.1 [− 0.2–0]	0.611
Inspiratory delay, ms	230 [200–290]	240 [180–290]	− 30 [− 43–9]	0.289
Ventilator insufflation time, s	1 [0.9–1.2]	0.8 [0.7–1.2]	− 0.1 [− 0.3–0.2]	0.519
Tidal volume				
mL	385 [322–485]	332 [237–398]	− 71 [− 176–2]	0.052
mL/kg of PBW	6.5 [5.5–8.2]	5.6 [4.1–7.2]	− 1.0 [− 3.0–0.0]	0.045
Respiratory rate				
Ventilator, breaths/min	26 [22–27]	35 [29–40]	7 [3–14]	0.007
Patient, breaths/min	41 [28–46]	39 [30–43]	− 2 [− 4–1]	0.264
Minute ventilation, L/min	9.1 [8.3–11.8]	11 [8.8–12.5]	0.7 [− 1.9–2.1]	0.791
Ventilatory ratio*	2.1 [1.41–2.3]	1.74 [1.29–2.65]	0.1 [− 0.1–0.5]	.0515
Pmus_peak_, cmH_2_O	9.1 [4.7–11.4]	11.6 [9.6–13.2]	3.1 [− 0.1–5.7]	0.06
Total PTPes, cmH_2_O/s/min	107 [79–131]	149 [129–170]	43 [1–89]	0.092
Effective PTPes, cmH_2_O/s/min	84 [70–104]	135 [127–161]	54 [20–84]	0.016
Ineffective PTPes, cmH_2_O/s/min	8 [3–30]	2 [1–6]	− 8 [− 28–− 2]	0.012
Intrinsic PEEP, cmH_2_O	3.9 [3–4.1]	5.6 [2.1–7.2]	0.5 [− 1.3–2.5]	0.380

PSV: Pressure support ventilation with the lowest tolerated pressure support level; PAV+: proportional assist ventilation with load-adjustable gain factors with the gain adjusted to target a peak muscle pressure between 5 and 10 cmH_2_O; the Δ (PAV+-PSV) column represents the median [interquartile range] of individual differences between PAV+ and PSV. MAP: mean arterial pressure; PBW: predicted body weight; Pmus_peak_: peak muscle pressure; PTPes: esophageal pressure–time product; PEEP: positive end-expiratory pressure. Note that intrinsic PEEP was measured as the drop in esophageal pressure before increase in flow, which could also capture expiratory muscles relaxation. Effective PTPes denotes PTPes recorded during respiratory efforts that effectively trigger an insufflation from the ventilator; ineffective PTPes denotes PTPes recorded during ineffective triggerings. * Ventilatory ratio is defined as (minute ventilation × PaCO_2_)/((predicted body weight/10) × 37.5))

Median duration of mechanical ventilation was 11 days [9–19]. Two patients were tracheostomized during the weaning process. According to the WIND classification [27] three patients had short weaning, four difficult weaning, four prolonged weaning, and one no weaning. Main reasons for difficult and prolonged weaning were weaning-induced pulmonary edema (*n* = 2), multiple ventilator-associated pneumonia (*n* = 1), need for continuous analgesia and sedation (e.g., pain related to necrotizing fasciitis; *n* = 3), and critical illness polyneuropathy and myopathy (*n* = 2). Three patients had prolonged duration of mechanical ventilation due to either the severity of the underlying disease (e.g., pancreatitis with multiple collections) or ICU-acquired complications. Two patients died before hospital discharge.

### Primary endpoint

**Fig. 2:**
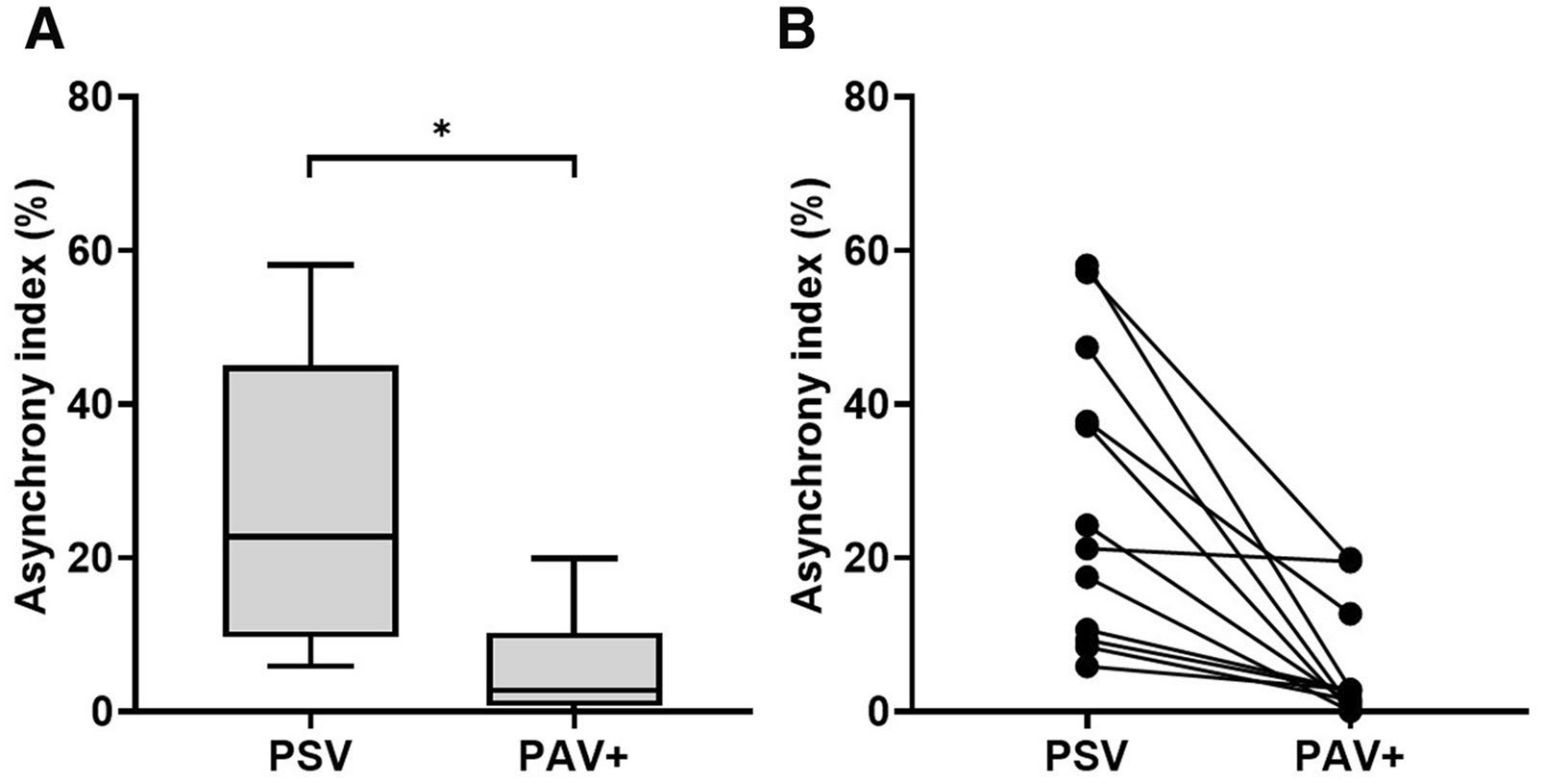
Asynchrony index under PSV and PAV+. *Physiological study. *Asynchrony index, defined as ineffective efforts/(effective efforts + ineffective efforts) under pressure support ventilation (PSV) with the lowest tolerated pressure support level and proportional assist ventilation with load-adjustable gain factors (PAV+) with the gain adjusted to target a peak muscle pressure between 5 and 10 cmH_2_O. **A** The box plots represent the asynchrony index (thick horizontal bar: median; extremities of the boxes: 25th and 75th percentiles; thin horizontal bars: fifth and 95th percentiles). * Denotes statistical significance. **B** Individual data

**Fig. 3:**
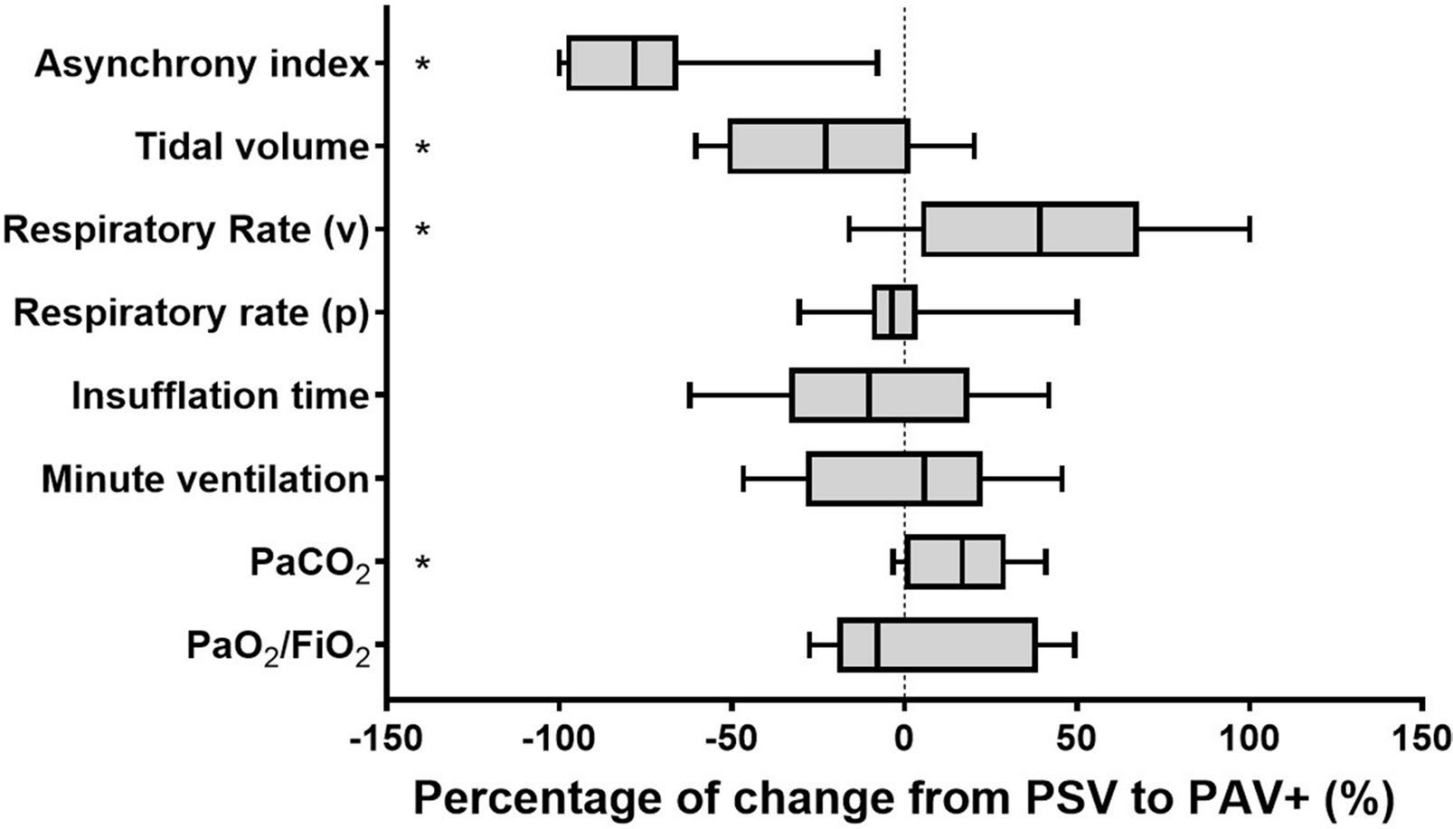
Ventilatory variables under PSV and PAV+. *Physiological study.* Relative changes, expressed as percentages, of ventilatory variables between pressure support ventilation (PSV) with the lowest tolerated pressure support level and proportional assist ventilation with load-adjustable gain factors (PAV+) with the gain adjusted to target a peak muscle pressure between 5 and 10 cmH_2_O. * Denotes statistical significance

The asynchrony index was significantly lower during PAV+ than during PSV with the lowest tolerated PSL (2.7% [1.0–5.4] vs*.* 22.7% [10.3–40.2], *p* < 0.001) (Fig. 2A). This corresponded to a relative decrease of 78% [66–96] of the asynchrony index with PAV+ (Fig. 3). Individual data analysis retrieved a reduction of asynchrony index with PAV+ in all patients (Fig. 2B). During PSV with the lowest tolerated PSL, nine patients (75%) exhibited an asynchrony index above 10%, the minimal asynchrony index recorded was 5.9% and the maximal 58.1%. During PAV+ with the gain adjusted according to the Pmus_peak_, three patients (25%) still experienced an asynchrony index above 10%, the nine remaining patients having an asynchrony index below 3%. The minimal asynchrony index during PAV+ was 0% (*n* = 2) and the maximal 19.9%.

### Secondary endpoints

As compared to PSV with the lowest tolerated PSL, PAV+ with the gain adjusted according to the Pmus_peak_ led to a significant decrease in tidal volume and a significant increase in ventilator’s respiratory rate (Table 3 and Fig. 3). Thus, the minute ventilation did not significantly differ between PSV and PAV+. As the patient’s respiratory rate did not significantly vary, the increase in ventilator’s respiratory rate was related to the decrease in ineffective triggering. The total respiratory effort, quantified by the mean of the PTPes, did not significantly vary between PSV and PAV+ (Table 3). However, the amount of effort lost in ineffective triggering significantly decreased during PAV+. Conversely, the part of effort that effectively triggered the ventilator and participated in tidal ventilation significantly increased during PAV+.

## Discussion

The main findings of our study are the following: PAV+ adjusted to target a desirable range of respiratory effort significantly reduced the incidence of refractory ineffective triggering, that was detected in about one in ten patients under PSV. Moreover, the magnitude of asynchrony reduction was clinically relevant as the asynchrony index decreased by almost 80%.

### Incidence of refractory ineffective triggering

In our study, 19% of patients assessed for eligibility exhibited a high incidence of ineffective efforts, which is consistent with previous findings reporting 12 to 45% of patients with an ineffective triggering index above 10% [1, 3, 4, 16]. Ineffective triggering usually occurs during PSV when increasing the PSL [6–8, 10]. In fact, increasing the PSL usually increases both the tidal volume and insufflation time [28–31]. Thus, the volume to exhale increases but the time to exhale decreases [32]. This can lead to dynamic hyperinflation [33–35], especially in patients with a high time constant of the respiratory system (e.g., patients with chronic obstructive pulmonary disease [COPD]) [35, 36]. In the meantime, the increase in PSL is accompanied by a decrease in respiratory drive and inspiratory effort [6, 28], which can become insufficient to overcome the intrinsic PEEP [33, 34, 37, 38], leading to the occurrence of ineffective triggering [1, 3, 5, 39, 40]. Therefore, ineffective triggering is frequently identified as a sign of over-assistance [5, 40], and Thille et al. showed that the most effective intervention to decrease or even suppress it was to decrease the PSL [11]. Some patients, however, still experience asynchrony despite optimization of the PSL. Indeed, in our series, in 21 out of the 36 patients with ineffective triggering, PSL adjustment alone failed to eliminate asynchrony. Since our study, the incidence of such refractory ineffective triggering was unknown. We retrieve that around 10% of the patients assessed in our series had refractory ineffective triggering. Moreover, 75% of them exhibited a high incidence (> 10%) of refractory asynchrony, up to 58.1%. This observed incidence most likely underestimates the true incidence of refractory asynchrony. In fact, it has been shown that ineffective triggering occurs in cluster [4], and can therefore be undetected in case of intermittent inspection of the ventilator's screen. As asynchronies have been reported to be associated with poor outcomes [2, 4], this significant proportion highlights the need to explore other ventilatory modalities in order to improve patient–ventilator interactions.

### Patients characteristics and effect of PAV+ on refractory asynchrony

As detailed above, the increase in time constant of the respiratory system is a characteristic that significantly favors the occurrence of ineffective triggering [35, 36, 39]. Most of our patients with refractory ineffective triggering under PSV also exhibited a high respiratory demand. In fact, 2/3 of them needed a PSL equal or above 16 cmH_2_O to maintain their respiratory effort (PTPes) within a normal range [24, 41], and their respiratory rate was generally high. This high respiratory demand may be explain by the impaired respiratory mechanics in some patients, seven out of the 12 included patients having a compliance below 40 mL/cmH_2_O (Table 2). Furthermore, three non-included patients with refractory asynchrony exhibited respiratory or hemodynamic instability, conditions usually accompanied by a need for increased respiratory support. These observations may suggest that the occurrence of refractory ineffective triggering could be a sign of poor tolerance of partial ventilatory support in some patients.

The use of PAV+ has been reported to be associated with less ineffective triggering than PSV [16, 18], but its effect on refractory asynchrony was unknown. It has also been shown that proportional modes, unlike PSV, protect against the occurrence of ineffective triggering when increasing, even significantly, the level of assistance [6, 8–10]. In addition, given the variability of patient inspiratory effort and respiratory load conditions over time, the ability of proportional mode to adapt to patient ventilatory demand better than PSV [42] may be useful when titration of the level of support is challenging, such as in the case of refractory ineffective triggering. Lastly, with PAV+, we previously reported that it was feasible to adjust the level of assistance in order to target a desirable range of respiratory effort [15], and thus to tailor the assistance on the patient’s need. This may explain why PAV+ with the gain adjusted according to the Pmus_peak_ significantly decreased or even suppressed refractory ineffective triggering. Of note, the intrinsic PEEP, as measured, was higher with PAV+ than with PSV, which may seem at odds with the pathophysiology of ineffective triggering described above. However, intrinsic PEEP was measured as the drop in esophageal pressure before the increase in flow, which could also capture expiratory muscles relaxation. Thus, it is possible that intrinsic PEEP has been overestimated during PAV+, as the respiratory effort tended to be higher with this mode. In addition, intrinsic PEEP may vary cycle to cycle and was by definition measured during triggered cycles and not during ineffective triggering, which may have led to an underestimation of mean intrinsic PEEP during PSV. In our study the PTPes tended to be higher with PAV+, but the tidal volume was significantly lower, suggesting that the method of gain titration resulted in a lower level of assistance than during PSV with the lowest tolerated PSL, which may have participated in the reduction in asynchrony. Whether such modifications in patient–ventilator interactions with PAV+ may be accompanied by a clinical benefit remains largely unknown and is currently being assessed in an ongoing multicenter clinical study (NCT02447692).

### Strength and limitations

This is a single-center study conducted in an expert team in the field of patient–ventilator interactions. Additionally, the pre-test probability of observing ineffective triggering is influenced by the proportion of patients prone to develop dynamic hyperinflation, as COPD patients (10% in our population). Our results may therefore not be generalizable to other centers. However, our medical ICU admits a wide variety of patients and previous work on asynchrony conducted in our site (1) was found reproducible in other centers (3). The screening did not involve every patient under PSV and we eventually explored a small sample size. Our results may therefore not apply to all patients experiencing refractory ineffective triggering under PSV. However, the number of screened patients remained large, and the careful physiological assessment led to remarkably consistent results across all patients. During PSV, the optimization of ventilator’s settings to suppress ineffective triggering only involved the PSL. As increase in insufflation time favors the occurrence of dynamic hyperinflation, increasing the cycling-off criterion to better match the patient’s neural inspiratory time may also be proposed to decrease the incidence of ineffective triggering [11]. PEEP was also kept constant during this study. It has been shown that PEEP personalization in order to help the respiratory muscles in overcoming the intrinsic PEEP is efficient to decrease the incidence of ineffective triggering [35]. However, in 11 out of our 12 included patients, the amount of intrinsic PEEP was below external PEEP. Additionally, the reported value of intrinsic PEEP may have been overestimated. In fact it was calculated as the drop in esophageal pressure before the increase in flow, which might have been influenced by expiratory muscle relaxation, especially during PAV+ where the measured intrinsic PEEP increased but the ineffective effort decreased. Of note, three out of the 21 patients with ineffective triggering had been maintained by their clinician in PSV despite severe hypoxemia or hemodynamic instability (Fig. 1). Lastly, during PAV+, the algorithm used to titrate the gain may have limited accuracy in estimating the muscle pressure, especially in case of intrinsic PEEP [43, 44]. This may explain slightly higher than expected Pmus_peak_ values during PAV+.

## Conclusions

Among patients with ineffective triggering under PSV, PSL adjustment failed to eliminate asynchrony in 58% of them. In these patients with refractory ineffective triggering, switching from PSV to PAV+ significantly reduced or even suppressed the incidence of asynchrony. Ongoing clinical study will assess whether such improvement in patient–ventilator interactions with PAV+ is accompanied by a clinical benefit.

## Supplementary Information

**Additional file 1.** On this schematic representation of flow, airway pressure (Paw) and esophageal pressure (Pes), insufflation time was defined as the time from the onset to the end of positive flow (**a**); inspiratory delay was defined as the time between the onset of the decrease in esophageal pressure and the beginning of the ventilator’s insufflation (**b**); intrinsic PEEP was defined as the esophageal pressure drop during the inspiratory delay (**c**); tidal volume was obtained by integrating the flow signal during insufflation (shaded area).

## Data Availability

The datasets used and/or analyzed during the current study are available from the corresponding author on reasonable request.

## Abbreviations

AHF: Acute heart failure
BMI: Body mass index
COPD: Chronic obstructive pulmonary disease
FiO_2_: Fraction of inspired oxygen
MAP: Mean arterial pressure
MV: Mechanical ventilation
PaCO_2_: Partial pressure of carbon dioxide
PaO_2_: Partial pressure of oxygen
PAV+: Proportional assist ventilation with load-adjustable gain factors
Paw: Airway pressure
PB: Puritan Bennett®
PEEP: Positive end-expiratory pressure
Pes: Esophageal pressure
Pmus: Muscle pressure
PSL: Pressure support level
PSV: Pressure support ventilation
PTP: Pressure–time product
PTPes: Esophageal pressure–time product
RASS: Richmond agitation-sedation scale
RR: Respiratory rate
SAPS II: Simplified acute physiology score II
SOFA: Sepsis-related Organ Failure Assessment
SpO_2_: Peripheral oxygen saturation
