# Supplementary material for: Refractory ineffective triggering during pressure support ventilation: effect of proportional assist ventilation with load-adjustable gain factors
Source: Ann Intensive Care. 2021 Oct 20;11:147. doi: 10.1186/s13613-021-00935-0 (PMC8527439; doi:10.1186/s13613-021-00935-0)
Supplement: Supplementary file 1 — Additional file 1. On this schematic representation of flow, airway pressure (Paw) and esophageal pressure (Pes), insufflation time was defined as the time from the onset to the end of positive flow (a); inspiratory delay was defined as the time between the onset of the decrease in esophageal pressure and the beginning of the ventilator’s insufflation (b); intrinsic PEEP was defined as the esophageal pressure drop during the inspiratory delay (c); tidal volume was obtained by integrating the flow signal during insufflation (shaded area). [file 13613_2021_935_MOESM1_ESM.docx]

**SUPPLEMENTAL DIGITAL CONTENT**

**Title**

**Proportional assist ventilation with load-adjustable gain factors decreases the incidence of refractory asynchrony**

**List of Authors**

Anne-Fleur HAUDEBOURG, Tommaso MARAFFI, Samuel TUFFET, François PERIER, Nicolas DE PROST, Keyvan RAZAZI, Armand MEKONTSO DESSAP, Guillaume CARTEAUX

**Additional methods (physiological study)**

The flow, airway and esophageal pressure signals were recorded during the last 10 minutes of each 20-minute period. Flow was recorded using a heated pneumotachograph AC137A-1 (Biopac Systems, Goleta, CA, USA) inserted between the Y piece of the ventilator circuit and the endotracheal tube and connected to a differential pressure transducer TSD160A (Biopac Systems, Goleta, CA, USA). Airway pressure was measured at the distal end of the circuit using a differential pressure transducer TSD160D (Biopac Systems, Goleta, CA, USA). Esophageal pressure was measured using an esophageal catheter (Nutrivent® catheter, Sidam, San Giacomo Roncole, Mirandola, Modena, Italia), whose balloon was inflated with 4 ml of air and connected to a differential pressure transducer TSD160C (Biopac Systems, Goleta, CA, USA). Appropriate placement of the esophageal balloon was verified using an occlusion test [1]. All signals were recorded at 1000 Hz using an analog/numeric data-acquisition system (MP150, Biopac systems, Goleta, CA, USA) and stored in a computer for subsequent analysis.

Ineffective efforts were identified from the combined analysis of the flow, airway and esophageal pressures signals as previously described [2]. Their frequency was expressed as the asynchrony index [2]. Inspiratory delay was defined as the time between the onset of the decrease in esophageal pressure and the beginning of the ventilator’s insufflation. The intrinsic PEEP was defined as the esophageal pressure drop during the inspiratory delay (figure 2). The insufflation time was defined as the time from the onset to the end of positive flow. The tidal volume was obtained by integrating the flow signal during insufflation. Muscle pressure and esophageal pressure time product (PTPes) was computed as previously described [3]. These five measurements were averaged on fifteen cycles.

REFERENCES

1. Baydur A, Behrakis PK, Zin WA, Jaeger M, Milic-Emili J. A simple method for assessing the validity of the esophageal balloon technique. Am Rev Respir Dis. 1982;126:788–91.

2. Thille AW, Rodriguez P, Cabello B, Lellouche F, Brochard L. Patient-ventilator asynchrony during assisted mechanical ventilation. Intensive Care Med. 2006;32:1515–22.

3. Mauri T, Yoshida T, Bellani G, Goligher EC, Carteaux G, Rittayamai N, et al. Esophageal and transpulmonary pressure in the clinical setting: meaning, usefulness and perspectives. Intensive Care Med. 2016;42:1360–73.

**Figure Legend**

**e-Figure 1. Main ventilatory measurements**

On this schematic representation of flow, airway pressure (Paw) and esophageal pressure (Pes), insufflation time was defined as the time from the onset to the end of positive flow (a); inspiratory delay was defined as the time between the onset of the decrease in esophageal pressure and the beginning of the ventilator’s insufflation (b); intrinsic PEEP was defined as the esophageal pressure drop during the inspiratory delay (c); tidal volume was obtained by integrating the flow signal during insufflation (shaded area).

**e-Figure 1**

**
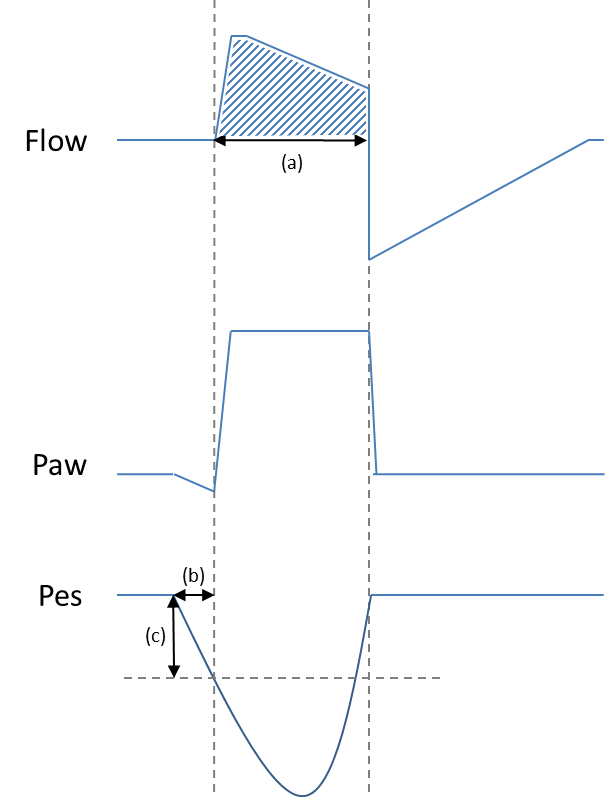
**
